# Plasma MIC-1 correlates with systemic inflammation but is not an independent determinant of nutritional status or survival in oesophago-gastric cancer

**DOI:** 10.1038/sj.bjc.6605532

**Published:** 2010-01-26

**Authors:** R J E Skipworth, D A C Deans, B H L Tan, K Sangster, S Paterson-Brown, D A Brown, M Hunter, S N Breit, J A Ross, K C H Fearon

**Affiliations:** 1 Clinical and Surgical Sciences (Surgery), University of Edinburgh, Royal Infirmary of Edinburgh, 51 Little France Crescent, Edinburgh EH16 4SA, UK; 2 St Vincent's Centre for Applied Medical Research, St Vincent's Hospital and University of New South Wales, Victoria Street, Sydney, New South Wales 2010, Australia

**Keywords:** macrophage inhibitory cytokine 1, oesophageal cancer, gastric cancer, cachexia, survival, nutrition

## Abstract

**Background:**

Macrophage inhibitory cytokine-1(MIC-1) is a potential modulator of systemic inflammation and nutritional depletion, both of which are adverse prognostic factors in oesophago-gastric cancer (OGC).

**Methods:**

Plasma MIC-1, systemic inflammation (defined as plasma C-reactive protein (CRP) of ⩾10 mg l^–1^ or modified Glasgow prognostic score (mGPS) of ⩾1), and nutritional status were assessed in newly diagnosed OGC patients (*n*=293). Healthy volunteers (*n*=35) served as controls.

**Results:**

MIC-1 was elevated in patients (median=1371 pg ml^–1^; range 141–39 053) when compared with controls (median=377 pg ml^–1^; range 141–3786; *P*<0.001). Patients with gastric tumours (median=1592 pg ml^–1^; range 141–12 643) showed higher MIC-1 concentrations than patients with junctional (median=1337 pg ml^–1^; range 383–39 053) and oesophageal tumours (median=1180 pg ml^–1^; range 258–31 184; *P*=0.015). Patients showed a median weight loss of 6.4% (range 0.0–33.4%), and 42% of patients had an mGPS of ⩾1 or plasma CRP of ⩾10 mg l^–1^ (median=9 mg l^–1^; range 1–200). MIC-1 correlated positively with disease stage (*r*^2^=0.217; *P*<0.001), age (*r*^2^=0.332; *P*<0.001), CRP (*r*^2^=0.314; *P*<0.001), and mGPS (*r*^2^=0.336; *P*<0.001), and negatively with Karnofsky Performance Score (*r*^2^=−0.269; *P*<0.001). However, although MIC-1 correlated weakly with dietary intake (r^2^=0.157; *P*=0.031), it did not correlate with weight loss, BMI, or anthropometry. Patients with MIC-1 levels in the upper quartile showed reduced survival (median=204 days; 95% CI 157–251) when compared with patients with MIC-1 levels in the lower three quartiles (median=316 days; 95% CI 259–373; *P*=0.036), but MIC-1 was not an independent prognostic indicator.

**Conclusions:**

There is no independent link between plasma MIC-1 levels and depleted nutritional status or survival in OGC.

Systemic inflammation has been linked with adverse survival in a variety of cancer types (Falconer *et al*, 1995; Scott *et al*, 2002; Hefler *et al*, 2008). This association could be explained by a variety of tumour-related phenomena, including enhanced tumour progression (Hefler *et al*, 2008), angiogenesis (Krzystek-Korpacka *et al*, 2008), and metastasis (Weinstein *et al*, 1984). However, the presence of systemic inflammation has also been linked with both hypermetabolism (Falconer *et al*, 1994) and reduced food intake (Fearon *et al*, 2006), two key components of the cachexia syndrome that is known to result in shortened survival in patients with advanced malignancy (Fearon *et al*, 2006). The mechanisms by which systemic inflammation arises in cancer patients are not established clearly. One hypothesis is that interaction between host and tumour cells within the tumour mass results in activation of peripheral blood mononuclear cells (PBMCs) (O’Riordain *et al*, 1999). The latter circulate to distant target organs in which enhanced cytokine/mediator production results in the generation of a systemic inflammatory response. Target organs include the liver (production of acute-phase proteins, e.g., C-reactive protein (CRP); O’Riordain *et al*, 1999), the brain (induction of anorexia; Buchanan and Johnson, 2007), and skeletal muscle (induction of protein degradation and net amino-acid mobilisation; Acharyya *et al*, 2005; Dejong *et al*, 2005). We have shown previously increased pro-inflammatory cytokine release by PBMCs in patients with elevated plasma CRP concentrations (O’Riordain *et al*, 1999). The ability of such PBMCs to induce acute-phase protein production from co-cultured human hepatocytes seemed to be IL-6 dependent (O’Riordain *et al*, 1999). In patients with oesophago-gastric cancer (OGC; in which systemic inflammation is associated with weight loss and shortened survival), we have previously shown that tumour IL-1*β* overexpression and chronic inflammatory cell infiltrate are independent factors influencing systemic inflammation (Deans *et al*, 2006). However, the precise role of various pro-inflammatory cytokines within human tumours in the generation of a systemic response is still incompletely understood.

Macrophage inhibitory cytokine-1 (MIC-1) is a divergent member of the transforming growth factor-*β* superfamily that is produced by macrophages in response to activation (Bootcov *et al*, 1997). MIC-1 is not expressed in most human tissues at basal conditions (except the placenta) but is expressed at high concentrations during inflammation and injury (Fairlie *et al*, 1999; Schober *et al*, 2001). MIC-1 is overexpressed by malignant melanoma cells and is associated with tumourigenicity (Boyle *et al*, 2009). High serum concentrations of MIC-1 have been observed in patients with pancreatic (Koopmann *et al*, 2004, 2006), gastric (Baek *et al*, 2009), and breast cancer (Welsh *et al*, 2003), and have been associated with adverse survival in colorectal cancer (Brown *et al*, 2003) and glioblastoma (Shnaper *et al*, 2009). In prostate cancer, high patient serum levels of MIC-1 have been associated with increased disease stage (Selander *et al*, 2007), docetaxel resistance (Zhao *et al*, 2009), and adverse survival (Brown *et al*, 2009). In prostate cancer bone metastases, MIC-1 induced osteoclast activation (Wakchoure *et al*, 2009). In mice bearing human prostate cancer xenografts, elevated MIC-1 concentrations were associated with marked weight, fat, and lean tissue loss that was mediated by decreased food intake and was reversed by an antibody to MIC-1 (Johnen *et al*, 2007). In addition, normal mice administered systemic MIC-1, and transgenic mice overexpressing MIC-1, showed hypophagia and reduced body weight (Johnen *et al*, 2007). In a small group of cachectic prostate cancer patients (*n*=26), serum MIC-1 concentrations were significantly associated with weight loss and weakly correlated with serum IL-6 (Johnen *et al*, 2007).

To determine whether MIC-1 is associated with inflammatory and nutritional status, we aimed to measure circulating concentrations of MIC-1 in a large cohort (*n*>250) of patients with OGC, a disease strongly associated with cachexia, and to analyse the relationships between MIC-1, systemic inflammation, nutritional status, and survival.

## Materials and methods

### Patients and controls

All patients provided written, informed consent, and the study was approved by the Lothian Research Ethics Committee. Patients with a new histological diagnosis of OGC were recruited (*n*=293; 198 males and 95 females). Whole blood was taken at diagnosis for plasma analysis of MIC-1 and CRP concentration. Staging was carried out according to the AJCC/UICC system (Sobin and Wittekind, 2002). The majority of patients (*n*=186) were followed up until death. A healthy control cohort (*n*=35; 25 males, 10 females, median age 29 years, range 24–85), composed of laboratory and hospital staff (*n*=28) and hospital patients undergoing minor operative procedures for benign conditions (*n*=7), was recruited for comparative MIC-1 analysis. Exclusion criteria for controls included recent weight change, ill-health, or underlying inflammatory illness (e.g., rheumatoid arthritis).

### Nutritional assessment

At recruitment, pre-illness stable weight was self-reported by patients and percentage weight loss was calculated (Deans *et al*, 2009). Height was measured using a wall-mounted stadiometer with the patient standing erect without shoes. Patients were weighed on spring balance scales without shoes and wearing light clothing. Mid-arm circumference (MAC) was measured at the mid-point between the acromion and olecranon processes. Triceps skinfold thickness (TSF) was measured with Harpenden skin callipers (Holtain, Crymych, UK). Mid-arm muscle circumference (MAMC) was calculated according to the formula: MAMC=MAC-[*π* × TSF]. Karnofsky performance score (KPS) was documented in all patients by the recruiting physician. As surrogates of dietary intake, dysphagia score (normal swallowing=0; dysphagia to solids=1; dysphagia to softened foods=2; dysphagia to liquids=3; and total dysphagia=4) and subjective diet score (normal dietary intake=1; reduced dietary intake=2; and poor dietary intake=3) were assessed in a subset of patients (*n*=188). Cachexia was defined as weight loss of ⩾10% when compared with pre-morbid weight. Control subjects did not undergo nutritional assessment beyond confirmation of weight stability.

### Total plasma MIC-1 concentration determination

Samples were examined in duplicate using a well-established sandwich ELISA as previously described (Moore *et al*, 2000). In brief, the mouse MAb 26G6H6 was used for antigen capture; and the sheep PAb 233-P was used for detection (Moore *et al*, 2000; Fairlie *et al*, 2001; Brown *et al*, 2002). The hMIC-1 plasma concentration was determined by reference to a standard curve constructed using recombinant hMIC-1 as the standard. All samples had duplicate values with a coefficient of variation (CV) of <10%. Assay performance was monitored additionally using standard diagnostic laboratory procedures.

### Assessment of systemic inflammation

Systemic inflammation was determined in two ways. In all patients, the presence of a plasma CRP concentration of ⩾10 mg l^–1^ was used to define an acute-phase protein response (APPR). In a subset of patients (*n*=197), plasma albumin concentration was determined, and thus calculation of a systemic inflammation-based score, the modified Glasgow Prognostic Score (mGPS) (McMillan, 2009), was performed. The mGPS was calculated as follows: patients with an elevated level of CRP (>10 mg l^–1^) were allocated a mGPS of 1 or 2, depending on the absence or presence of hypoalbuminaemia (<35 g l^–1^), whereas patients showing no elevated level of CRP (⩽10 mg l^–1^) were allocated a mGPS of 0. Plasma CRP and albumin concentrations were assayed using automated methods on Olympus AU 2700 and Olympus 640 analysers, respectively (Olympus Diagnostica GmbH (Irish Branch), Lismeehan, Ireland), in the Department of Clinical Chemistry, Royal Infirmary of Edinburgh (fully accredited by Clinical Pathology Accreditation (UK) Ltd). Appropriate IQC were included, with CVs typically 3.4% at concentrations of <15 mg l^–1^and 1.6% at 80 mg l^–1^ for CRP, and <3.0% at all concentrations for albumin.

### Statistical analysis

All statistical analyses were performed using Statistical Package for Social Services version 13.0 (SPSS 13.0; Chicago, IL, USA). Plasma MIC-1 data are presented as box plots. Mild outliers (MIC-1 concentration more than 1.5 times the interquartile range (IQR) above the third quartile) are represented as circles and extreme outliers (MIC-1 concentration more than 3 times the IQR above the third quartile) are presented as stars. For visual clarity, the *y* axes are limited to a maximum MIC-1 concentration of 5000 or 8000 pg ml^–1^. Differences between the distribution functions of data for three or more subject groups were determined using Kruskal–Wallis test (shown in text and figure legends). Subsequent analysis for determining differences between any two groups was determined using Mann–Whitney U test (shown in figures). All quoted *P*-values are two tailed. Correlation analysis was performed using non-parametric Spearman's rank correlation coefficient. Linear regression was used for analysing the relationship between CRP and MIC-1. Significance levels for the explanatory variables of interest were 0.10 to enter stepwise into the model. Survival analyses were performed on those patients followed until death using Kaplan–Meier plots and Cox's proportional hazards models. For construction of the latter model, treatment regimen was defined as either surgery with curative intent, radical chemo/radiotherapy with curative intent, chemo/radiotherapy with palliative intent, or nil. Statistical significance was set at *P*<0.05 level.

## Results

### Patient demographics

Oesophago-gastric cancer patients showed a median weight loss of 6.4% (range 0.0–33.4% Table 1), and 34% of patients had lost ⩾10% body weight, consistent with significant cachexia. Furthermore, median BMI, MAMC, and TSF measures were lower than those reported in healthy elderly populations (Corish and Kennedy, 2003). In all, 42% of patients (*n*=123) showed plasma CRP concentrations of ⩾10 mg l^–1^ consistent with the presence of an APPR. Of the assessed patients, 29.9% (59 out of 197) had a mGPS of 1, and 13.7% (27 out of 197) had a mGPS of 2.

### Plasma MIC-1 concentrations are elevated in oesophago-gastric cancer

**Figure 1:**
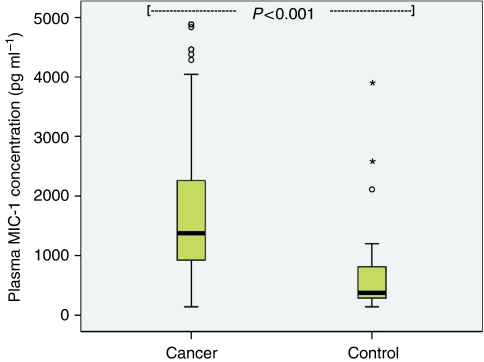
Box plot showing increased plasma MIC-1 concentration in oesophago-gastric cancer patients when compared with the controls (*P*<0.001).

**Figure 2:**
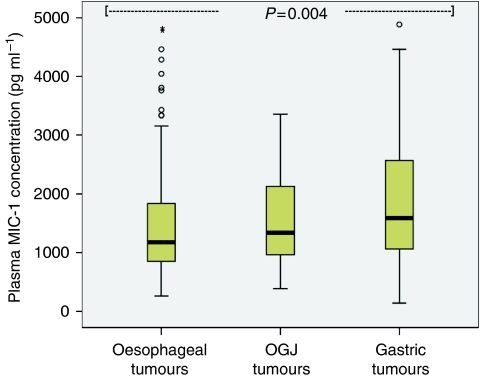
Box plot showing increasing plasma MIC-1 concentration with increasing distal situation of the primary tumour (*P*=0.015).

**Figure 3:**
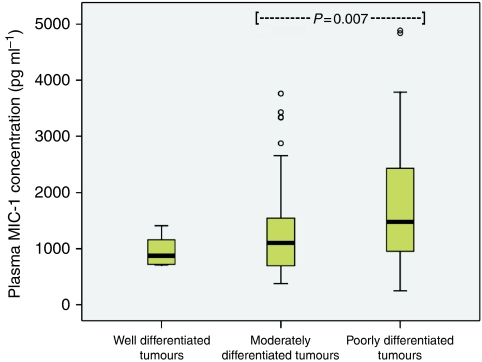
Box plot showing increasing plasma MIC-1 concentration with worsening tumour grade (*P*=0.010).

**Figure 4:**
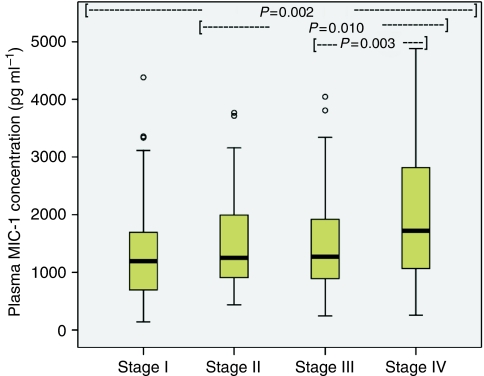
Box plot showing increasing plasma MIC-1 concentration with worsening disease stage (*P*=0.002).

**Figure 5:**
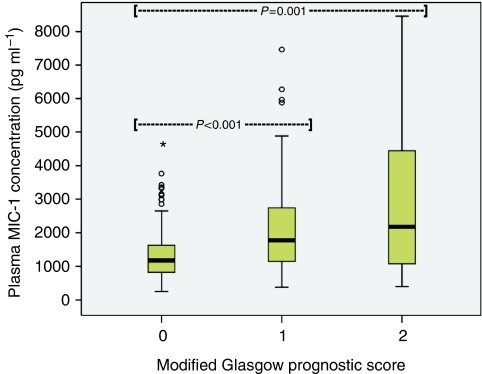
Box plot showing increasing plasma MIC-1 concentration with increasing modified Glasgow Prognostic Score (*P*<0.001).

Plasma MIC-1 was elevated in OGC patients (median 1371 pg ml^–1^; range 141–39 053) when compared with controls (median 377 pg ml^–1^; range 141–3786; *P*<0.001; Figure 1). Patients with gastric tumours (median 1592 pg ml^–1^; range 141–12 643) showed higher MIC-1 concentrations than patients with oesophago-gastric junction (OGJ; median 1337 pg ml^–1^; range 383–39 053) and oesophageal tumours (median 1180 pg ml^–1^; range 258–31 184; *P*=0.015; Figure 2). Patients with poorly differentiated tumours (median 1480 pg ml^–1^; range 245–9000) showed higher MIC-1 concentrations than patients with moderately differentiated (median 1103 pg ml^–1^; range 378–6646) and well-differentiated tumours (median 875 pg ml^–1^; range 710–1407; *P*=0.010; Figure 3). Plasma MIC-1 concentration also increased with worsening disease stage (Figure 4) and increasing mGPS (Figure 5).

### Relationship of MIC-1 with systemic inflammation and nutritional status

Plasma MIC-1 concentration correlated positively with disease stage (*r*^2^=0.217; *P*<0.001), patient age (*r*^2^=0.332; *P*<0.001), CRP (*r*^2^=0.314; *P*<0.001), and mGPS (*r*^2^=0.336; *P*<0.001), and correlated negatively with KPS (*r*^2^=−0.269; *P*<0.001; Table 2). Plasma MIC-1 also correlated weakly with diet score (*r*^2^=0.157; *P*=0.031) but did not correlate with dysphagia score or any of the nutritional and anthropometric parameters measured. However, there was a small but significant increase (18.9%) in plasma MIC-1 between patients who had lost ⩾10% weight (median 1493 pg ml^–1^; range 258–31 184) compared with those who had not (median 1256 pg ml^–1^; range 141–39 053; *P*=0.036). In contrast, both CRP (*r*^2^=0.247; *P*<0.001) and mGPS (*r*^2^=0.280; *P*<0.001) correlated with weight loss, and mGPS also correlated negatively with MAMC (*r*^2^=−0.318; *P*<0.001; Table 2). Furthermore, there was a highly significant increase in CRP and mGPS between patients who had lost ⩾10% weight compared with those who had not (median CRP 16.5 *vs* 6.0 mg l^–1^; median mGPS 1 *vs* 0; *P*=0.001 for both tests).

**Figure 6:**
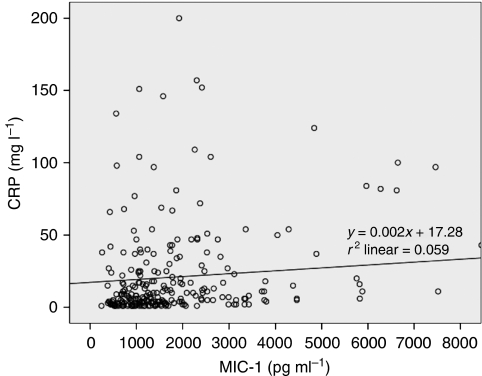
Dot plot of MIC-1 *vs* CRP (linear *r*^2^=0.059).

The relationship between MIC-1 and CRP was not linear, as dot-plots showed wide variance of CRP with increasing MIC-1 (Figure 6). However, assuming linearity, regression analysis showed that MIC-1 accounted for 5.9% of the variation in plasma CRP (*P*<0.001). In a regression model of CRP (incorporating MIC-1, disease stage, tumour grade, percentage weight loss, diet score and KPS, i.e., those factors found to correlate with CRP), plasma MIC-1 concentration (*P*=0.003), diet score (*P*=0.001), and tumour grade (*P*=0.033) were significant determinants (*r*^2^=0.179; Table 3). In a regression model of percentage weight loss (incorporating disease stage, mGPS, diet score, and dysphagia score), all four factors were significant determinants (*r*^2^=0.259; Table 3). Plasma MIC-1 concentration did not qualify to enter the model.

### Relationship of MIC-1 with survival

**Figure 7:**
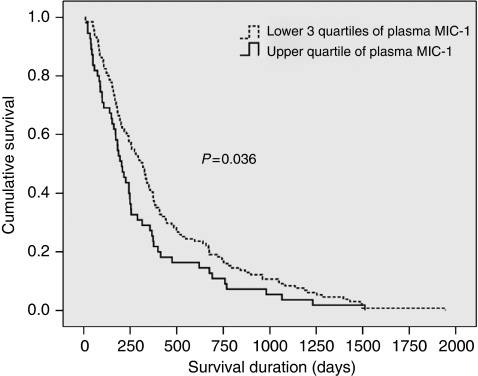
Kaplan–Meier plot of survival of oesophago-gastric cancer patients according to plasma MIC-1 concentration. Patients with MIC-1 concentrations in the upper quartile showed worsened survival (median 204 days; 95% CI 157–251) when compared with patients with MIC-1 concentrations in the lower three quartiles (median 316 days; 95% CI 259–373; *P*=0.036, log-rank test).

Patients with plasma MIC-1 concentrations in the upper quartile (>2270 pg ml^–1^) showed worsened survival (median 204 days; 95% CI 157–251) when compared with patients with MIC-1 concentrations in the lower three quartiles (median 316 days; 95% CI 259–373; *P*=0.036; log-rank test; Figure 7). In Cox's proportional hazards model (incorporating MIC-1, CRP, percentage weight loss, disease stage, tumour grade, patient age, dysphagia score, diet score, and treatment regimen), disease stage (*P*<0.001), treatment regimen (*P*=0.003), CRP (*P*=0.034), and percentage weight loss (*P*=0.002), but not plasma MIC-1 concentration, were significant determinants of survival (Table 4). Substitution of mGPS for CRP within the model revealed stage, treatment regimen, and weight loss as the only significant determinants.

## Discussion

This study shows that plasma MIC-1 concentrations are elevated in OGC patients when compared with controls. Furthermore, increasing plasma MIC-1 concentrations are associated with indicators of poor patient prognosis, including tumour grade and stage. Elevated circulating concentrations of MIC-1 have also been associated with poor prognostic indicators in other cancer types, including prostate cancer (Selander *et al*, 2007; Brown *et al*, 2009), colorectal cancer (Brown *et al*, 2003), and glioblastoma (Shnaper *et al*, 2009).

In this study, plasma MIC-1 also correlated significantly with mGPS and plasma CRP concentration, suggesting that MIC-1 might have a role in the aetiology of systemic inflammation in OGC (although linear regression implies that this may be a minor effect). However, MIC-1 concentrations do not correlate with any measured nutritional or anthropometric parameters, including patient weight loss. In a recent cohort of 220 patients with OGC, we have shown by multiple regression analysis that plasma CRP, stage of disease, and dietary intake are independent variables in determining the degree of weight loss (Deans *et al*, 2009). These results are confirmed by this study. Given such importance of systemic inflammation, disease stage, and dietary intake in the genesis of weight loss in OGC patients, it is somewhat surprising that although MIC-1 concentrations correlated with all three of these variables in this study, there was no correlation with weight loss. However, MIC-1 levels were slightly elevated in patients with ⩾10% weight loss when compared with patients without, suggesting that MIC-1 may have a role in the maintenance, rather than the initiation, of weight loss.

Studies in mice with xenografted prostate tumours have suggested that the mechanism underlying MIC-1-induced weight loss is hypophagia caused by reduced neuropeptide Y expression and increased pro-opiomelanocortin expression in the hypothalamic arcuate nucleus (Johnen *et al*, 2007). In patients with OGC, a number of primary and secondary causes of reduced dietary intake are at work simultaneously, including dysphagia (McKernan *et al*, 2008), early satiety (Davis *et al*, 2006), chronic nausea (McKernan *et al*, 2008), alterations in circulating neuroendocrine hormones (e.g., ghrelin (Isomoto *et al*, 2005) and leptin (Huang *et al*, 2005; Zhao *et al*, 2007)), hypogeusia/hyposmia (Plata-Salaman, 2000), and cytokine-induced central anorexia (Plata-Salaman, 2001; Turrin *et al*, 2004). Assuming that MIC-1 may be a modulator of appetite in humans, the complex constellation of additional factors also controlling food intake may explain why plasma MIC-1 concentrations did not correlate with nutritional status in this study. Future studies of MIC-1 that record accurately calorific intake in OGC patients are required to elucidate the anorectic effect of different factors.

Another proposed mechanism of MIC-1-induced weight loss is through paracrine effects on adipocytes (Ding *et al*, 2009). Recombinant MIC-1 stimulates adiponectin secretion by human adipocytes, thus potentially negatively regulating body fat mass (Ding *et al*, 2009). However, in this study, MIC-1 did not correlate with one measure of body fat, namely TSF. Furthermore, previous studies have proven that the relationship between MIC-1 and fat mass is clearly not understood fully, as both obesity and type II diabetes mellitus are associated with increased circulating levels of MIC-1 (Dostálová *et al*, 2009). However, the latter observation might be consistent with a role for MIC-1 in the aetiology of systemic inflammation.

An alternative hypothesis for explaining the lack of association between plasma MIC-1 and nutritional status in OGC is that circulating MIC-1 concentrations are simply not elevated sufficiently to overcome regulatory mechanisms and induce cachexia. In patients with prostate cancer and cachexia, serum MIC-1 concentrations were significantly higher (mean 12 416±s.d. 10 235 pg ml^–1^) (Johnen *et al*, 2007) than those measured in the present cohort of OGC patients. Furthermore, in mice xenografted with prostate tumours overexpressing human MIC-1, only animals with serum concentrations of >8500 pg ml^–1^ showed clinically significant levels of weight loss (Johnen *et al*, 2007). Of the OGC patients in this study, only 5 (1.7%) showed plasma concentrations of MIC-1 >8500 pg ml^–1^. In these individuals, plasma CRP was also elevated (median 58 mg l^–1^; range 16–92) but there was no significant reduction in BMI (median 30.1 kg m^–2^; range 21.7–32.0) or increase in weight loss (median 6.5% range 0.0–12.5) compared with the rest of the group. The reasons for low circulating MIC-1 concentrations in OGC patients may include low levels of activation of MIC-1 through p53-dependent mechanisms (Li *et al*, 2000; Albertoni *et al*, 2002; Yang *et al*, 2003), as many oesophago-gastric tumours show p53 deletion, mutation, and loss of heterozygosity (Huang *et al*, 1992; Renault *et al*, 1993).

In this study, elevated plasma MIC-1 was associated with worsened survival on univariate but not multivariate analysis. Such findings may vary depending on the definition of normal MIC-1 concentration used. Other studies that have analysed relatively large cohorts of healthy controls have suggested that the upper limit of normal plasma MIC-1 concentration lies between 1070 and 1600 pg ml^–1^ (Li *et al*, 2000; Brown *et al*, 2003; Koopmann *et al*, 2004, 2006), which is lower than the definition used in this study. However, the present data also suggest that plasma MIC-1 concentration may increase with patient age, thus implying that the normal range is not static. A lack of age-matched healthy controls could be considered a disadvantage in both previous studies and this study.

The variation in plasma MIC-1 concentrations observed between different tumour types, grades, and stages implicates a tumour-specific mechanism in the induction of MIC-1 expression. MIC-1 is often secreted in an unprocessed propeptide form that regulates the balance between extracellular matrix stores and circulating mature MIC-1 (Bauskin *et al*, 2005). The absence of propeptide in xenograft animal tumour models is associated with a 20-fold increase in serum MIC-1 (Bauskin *et al*, 2005). In low-grade localised prostate cancer, the level of proMIC-1 stromal stores was the best predictor of future disease relapse when compared with other clinicopathological variables (Bauskin *et al*, 2005). The mechanisms surrounding the processing and extracellular storage of MIC-1 may represent one explanation for the observed differences in plasma MIC-1 between different tumour variables.

In conclusion, although plasma MIC-1 correlates with tumour grade, disease stage, dietary intake, and systemic inflammation, it does not seem to mediate weight loss or nutritional depletion significantly in OGC.

## Figures and Tables

**Table 1:** Demographics, nutritional status, and plasma concentrations of inflammatory mediators of the oesophago-gastric cancer patients. Data are presented as medians with ranges.

	**Patients (*n*=293)**
*Male:female*	198 : 95
*Age* (years)	70 (26–91)
	
*Tumour site*	
Oesophageal	139
OGJ	51
Gastric	103
	
*Histology*	
Adenocarcinoma	242
Squamous	43
Undifferentiated	6
Neuroendocrine	2
	
*Disease stage*	
I	36
II	45
III	106
IV	97
Unknown	46
	
*Body mass index* (kg m^–2^)	24.6 (13.9–46.7)
*Weight loss* (% loss of pre-morbid weight)	6.4 (0.0–33.4)
*Mid-arm muscle circumference* (cm)	23.8 (15.6–32.10)
*Triceps skin-fold thickness* (mm)	12.0 (3.0–52.0)
*KPS*	90 (30–100)
*CRP* (mg l^–1^)	9.0 (1.0–200.0)
*MIC-1* (pg ml^–1^)	1246.5 (140.7–39 052.9)

Abbreviations: CRP=C-reactive protein; KPS=Karnofsky performance score; MIC-1=macrophage inhibitory cytokine-1.

**Table 2:** Correlations between inflammatory mediators and nutritional status

**Inflammatory mediator**	**Positive correlates**	***r* ^2^**	***P*-value**	**Negative correlates**	***r* ^2^**	***P*-value**
MIC-1	CRP	0.314	<0.001	Albumin	−0.316	<0.001
	mGPS	0.336	<0.001	KPS	−0.269	<0.001
	Age	0.332	<0.001			
	Tumour grade	0.234	0.002			
	Stage	0.217	<0.001			
	Diet score	0.157	0.031			
						
CRP	MIC-1	0.314	<0.001	Albumin	−0.489	<0.001
	Tumour grade	0.341	<0.001	KPS	−0.257	<0.001
	Stage	0.220	<0.001			
	% Weight loss	0.247	<0.001			
	Diet score	0.265	<0.001			
						
mGPS	MIC-1	0.336	<0.001	MAMC	−0.318	<0.001
	Tumour grade	0.268	0.001	KPS	−0.362	<0.001
	Stage	0.234	0.001			
	% Weight loss	0.280	<0.001			
	Diet score	0.262	<0.001			

Abbreviations: CRP=C-reactive protein; KPS=Karnofsky performance score; MAMC=mid-arm muscle circumference; mGPS=modified Glasgow Prognostic Score; MIC-1=macrophage inhibitory cytokine-1.

**Table 3:** Results of the regression models of CRP (*r*^2^=0.179) and percentage weight loss (*r*^2^=0.259) demonstrating significant determinants

		**Unstandardised coefficients**		**Standardised coefficients**			**95% CI for B**	
**Model**	**Factor**	**B**	**Standard error**	**β**	***t***	***P-*value**	**Lower boundary**	**Upper boundary**
CRP	(Constant)	−35.759	13.387		−2.671	0.008	−62.213	−9.305
	MIC-1	0.005	0.002	0.231	3.001	0.003	0.002	0.008
	Diet score	12.613	3.670	0.258	3.437	0.001	5.361	19.864
	Tumour grade	9.977	4.628	0.165	2.156	0.033	0.832	19.123
								
% Weight loss	(Constant)	−3.788	2.107		−1.798	0.074	−7.946	0.370
	Diet score	2.871	1.066	0.221	2.693	0.008	0.767	4.975
	mGPS	2.491	0.835	0.205	2.983	0.003	0.843	4.140
	Stage	1.459	0.639	0.161	2.284	0.024	0.198	2.719
	Dysphagia score	1.182	0.583	0.155	2.028	0.044	0.031	2.333

Abbreviations: CI=confidence interval; CRP=C-reactive protein; mGPS=modified Glasgow Prognostic Score; MIC-1=macrophage inhibitory cytokine-1.

**Table 4:** Results of the Cox's proportional hazards model demonstrating significant determinants of oesophago-gastric cancer patient survival

							**95% CI**	
	**B**	**SE**	**Wald**	**d.f.**	***P-*value**	**Hazard ratio**	**Lower boundary**	**Upper boundary**
Stage	0.682	0.177	14.902	1	<0.001	1.979	1.399	2.798
Treatment regimen			13.934	3	0.003			
CRP	0.006	0.003	4.498	1	0.034	1.006	1.000	1.012
% Weight loss	0.038	0.013	9.232	1	0.002	1.039	1.014	1.065

Abbreviations: CI=confidence interval; CRP=C-reactive protein.
